# Prediction of the Impact of Deleterious Nonsynonymous Single Nucleotide Polymorphisms on the Human *RRM2B* Gene: A Molecular Modeling Study

**DOI:** 10.1155/2020/7614634

**Published:** 2020-07-25

**Authors:** Chaimaa Ait El Cadi, Al Mehdi Krami, Hicham Charoute, Zouhair Elkarhat, Najat Sifeddine, Hamid Lakhiari, Hassan Rouba, Abdelhamid Barakat, Halima Nahili

**Affiliations:** ^1^Laboratory of Genomics and Human Genetics, Institut Pasteur du Maroc, 20360 Casablanca, Morocco; ^2^Laboratory of Biosciences, Functional Integrated and Molecular Exploration-LBEFIM-, Biology Department, Faculty of Sciences and Technics of Mohammedia, University of Hassan II, Mohammedia 28806, Morocco

## Abstract

*RRM2B* gene encodes ribonucleoside-diphosphate reductase subunit M2 B, the p53-inducible small subunit (p53R2) of ribonucleotide reductase (RNR), an enzyme catalyzing dNTP synthesis for mitochondrial DNA. Defects in this gene may cause severe mitochondrial disease affecting mainly the nervous system. This study is aimed at examining the effect of deleterious nonsynonymous SNP (nsSNP) on the structure of the RRM2B protein, using a variety of prediction tools followed by a molecular modeling analysis. After using 13 algorithms, 19 nsSNPs were predicted deleterious. Among these variants, 18 decreased the protein stability and 16 were localized in very highly conserved regions. Protein 3D structure analysis showed that 18 variants changed amino acid interactions. These results concur with what has been found in experimental trials; 7 deleterious nsSNPs were previously reported in patients suffering from genetic disorders affecting the nervous system. Thus, our study will provide useful information to design more efficient and fast genetic tests to find *RRM2B* gene mutations.

## 1. Introduction

Mitochondria are semiautonomous, self-reproducing organelles that occur in the cytoplasm of all cells of most eukaryotes. In that manner, a mitochondrial function can be provided by either mitochondrial or nuclear DNA; therefore, any disruption of both genetic materials can lead to severe mitochondrial diseases [1]. Human ribonucleotide reductase (RNR) is a heterotetramer enzyme that catalyzes the synthesis of dNTPs by direct reduction of ribonucleoside diphosphates to deoxyribonucleoside diphosphates required for DNA replication; it consists of two subunits: a large catalytic subunit called R1 and a small subunit called R2 [2, 3]. Actually, cells have two forms of the R2 subunit, a form regulated by the cell cycle expressed abundantly at the S phase and a second form regulated by the p53 tumor suppressor protein called p53R2.

This form is necessary for the synthesis and repair of mitochondrial DNA (mtDNA) inside nonproliferative cells [3, 4]. Indeed, this form is encoded by a gene known as ribonucleoside diphosphate reductase subunit M2 B or *RRM2B* located on chromosome 8 in position *8q22.3* [5].

Mutations within the *RRM2B* gene result in a variety of inherited mitochondrial diseases either recessive or dominant and can be divided into 2 major types: diseases characterized by mtDNA depletion and diseases characterized by multiple deletions of the mtDNA. mtDNA depletion, where inheritance is autosomal recessive, is generally characterized by severe multisystemic manifestations such as encephalomyopathy with proximal renal tubulopathy usually fatal in early age [5–10]. However, multiple mtDNA deletions cause tissue-specific cytochrome c oxidase (COX) deficiency. Inheritance can be either autosomal recessive with progressive external ophthalmoplegia (PEO) and multisystem involvement manifesting during early childhood/adulthood or autosomal dominant with less severe manifestations appearing during late adulthood [11–15]. We can also mention Kearns-Sayre syndrome (KSS) as one of the rare phenotypes that may occur due to mutations in the *RRM2B* gene. *RRM2B* gene variants can be found within all exons, but exon 9 is specifically considered as a mutation hotspot, since variations in this region are responsible for a truncated protein presumed to cause a dominant-negative or gain-of-function effect on the heterotetrameric structure of the RNR enzyme [11, 12].

There is currently a large number of *in silico* tools used to predict the structural impact caused by amino acid (aa) changes in a given molecule. These tools are not very accurate; however, they can still be used as an initial filter of potentially deleterious changes. Subsequently, we can first resort to molecular modeling which allows us to observe the impact of these changes on the protein 3D structure. Then, advanced analyses such as molecular dynamics simulation can be used to better assess the impact caused by these variants. However, the use of such a method requires a large computing power.

In this study, we will investigate the impact of nonsynonymous single nucleotide polymorphisms (nsSNPs) on the *RRM2B* protein, followed by molecular modeling analysis. This will allow us to evaluate the effect of potentially deleterious variations on the protein structure.

## 2. Data and Methods

### 2.1. Data and Sequence Extraction

The sequence of the *RRM2B* protein has been extracted in Fasta format from UniProt (ID: Q7LG56) (https://www.uniprot.org/uniprot/Q7LG56). Meanwhile, the SNPs were generated from NCBI's dbSNP database (https://www.ncbi.nlm.nih.gov/snp/).

### 2.2. Most Damaging Variant Identification

In order to have an idea about the impact of nsSNPs on the structure of the *RRM2B* protein, we used the following algorithms: SIFT [16], POLYPHEN [17], SNAP [18], PhD-SNP [19], Condel [20], PROVEAN [21], M-Cap [22], LRT [23], Mutation Assessor [24], Mutation Taster [25], PredictSNP [26], MAPP [27], and PANTHER [28]. Similarly to other studies [29], we maintained the standard cutoff or the threshold scores of all prediction tools to select the most deleterious amino acid changes on the protein function.

### 2.3. Stability and Conservation Analysis

I-Mutant (http://folding.biofold.org/i-mutant/) is a stability prediction software that measures the degree of protein destabilization and gives the predicted free energy change value DDG, which is the Gibbs free energy value from the mutated protein minus the Gibbs free energy value from the wild type expressed in Kcal/mol. The software judges an SNP “Increasing” the stability of the protein if the DDG value is superior to 0 or “Decreasing” it if the DDG value is below 0.

Mupro (http://mupro.proteomics.ics.uci.edu/) is also another stability prediction web server that uses support vector machines and neural networks, to predict the impact of a single site amino acid mutation on protein stability. This tool provides a prediction reliability score between -1 and 1.

Conservation analysis was conducted by ConSurf (http://consurf.tau.ac.il/), a web server identifying the functional regions of a protein by evaluating the degree of its conservation.

We also attempted to examine the exact number of conserved residues upon the *RRM2B* protein sequence, using the Structurally Conserved Region (SCR) prediction web server (http://prodata.swmed.edu/scr_prediction/index.php).

### 2.4. Molecular Modeling of Native and Variant Forms of the RRM2B Protein

For the purpose of having a clear picture of the effect of each variant on the structure of *RRM2B*, we need the three-dimensional structure (3D) of native protein and its different mutated forms. This was done by homology modeling using the SWISS-MODEL server [30, 31].

### 2.5. Sequence Visualization and Interaction Change Observation

After obtaining the PDB files of the 3D models, YASARA software [32] was used to visualize and evaluate possible changes in the interactions between amino acids comparing to the native model, using the various features and tools available.

## 3. Results

### 3.1. SNP's Distribution

**Figure 1:**
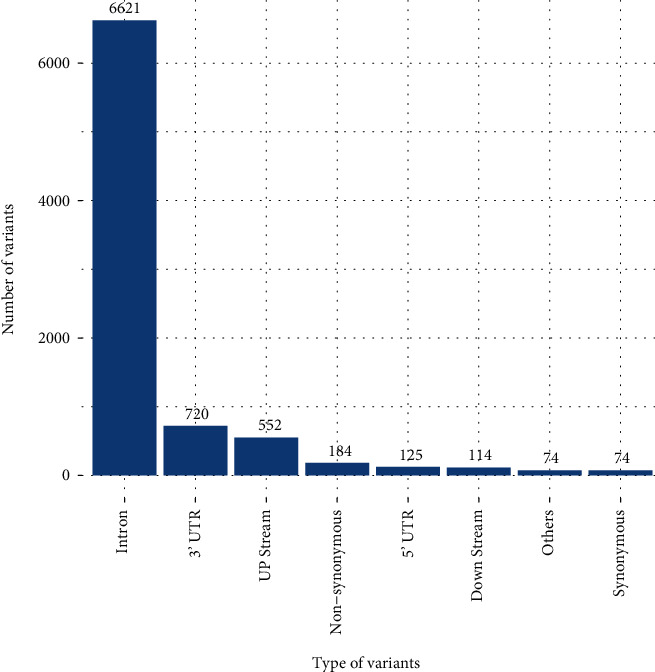
Distribution of SNPs present in the *RRM2B* gene.

During this study, we managed to extract a total of 8464 SNPs of the *RRM2B* gene using the dbSNP database of NCBI. 6621 of these SNPs are in the intron region, 720 in the 3′ UTR region, 552 upstream, 184 nonsynonymous, 125 in the 5′ UTR region, 114 downstream, 74 synonymous, and 74 others (Figure 1). This study focuses only on nonsynonymous SNPs.

### 3.2. Deleterious nsSNPs of the RRM2B Gene

The results of the 13 prediction algorithms showed that 19 among 184 nsSNPs were deleterious (Table 1). For the majority of prediction software, 1 is the higher score and 0 is the lower score, except for the SIFT tool where the higher score is 0 and the lower score is 1.

### 3.3. Stability Analysis

To investigate the effect of each of the 19 nsSNPs on the stability of the *RRM2B* protein, two web tools were used: I-Mutant and Mupro. According to the DDG values, only 1 nsSNP (Y124C) increased the stability of the *RRM2B* protein according to I-Mutant prediction, while for Mupro, all the 19 nsSNPs decreased the protein stability (Table 2).

### 3.4. Conservation Analysis

According to the conservation scale color code, we can see that the *RRM2B* protein sequence contains a high proportion of conserved regions, which make this protein more vulnerable for structural property alterations. These observations were confirmed through the examination of the exact number of conserved residues upon the *RRM2B* protein sequence, using the SCR prediction web server. The results showed that the *RRM2B* protein contains a total of 225 (out of 351) conserved residue representing 64% of the whole protein sequence (Table 3).

Using the ConSurf server, all the 19 nsSNP residues are predicted to be located in conserved regions of the *RRM2B* protein, representing exposed or buried and/or structural or functional residues. It is to mention that a structural residue is an amino acid that provides a structural framework to the protein, while a functional residue is an amino acid that mediates interactions of the protein with other biomolecules.

According to the conservation results, 16 nsSNPs were found to be localized in very highly conserved regions (9 on the conservation scale); 9 residues were predicted to be both exposed and functional, whereas 7 amino acids were buried and structural.

**Figure 2:**
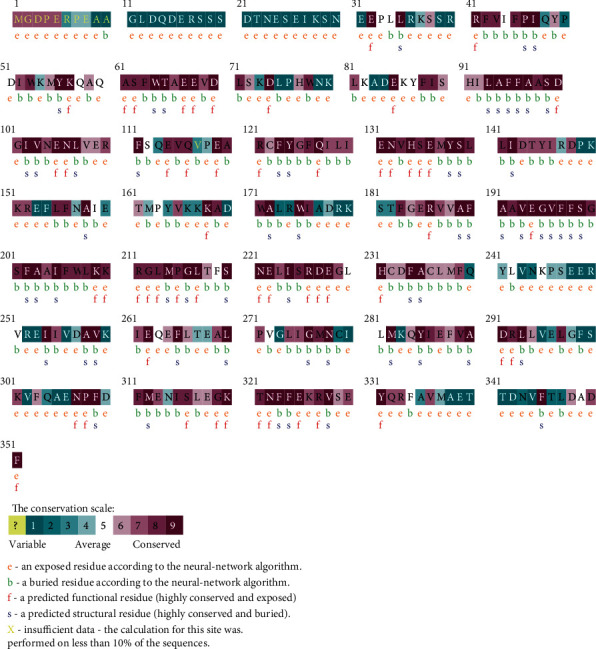
Conservation results of the *RRM2B* protein provided by the ConSurf web server.

Two buried nsSNPs were located in a conserved region (8 on the conservation scale). The remaining SNP was found in a moderately conserved region (7 on the conservation scale) (Figure 2).

### 3.5. Molecular Modeling

**Figure 3:**
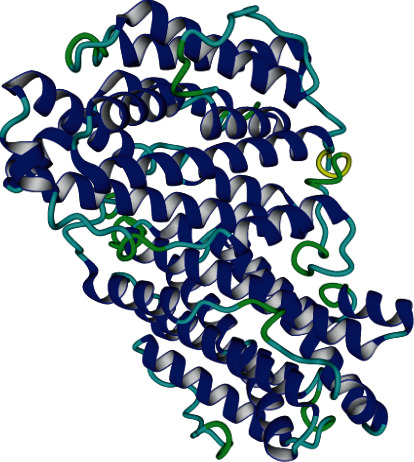
The RRM2B protein structure predicted using the SWISS-MODEL server.

The SWISS-MODEL server generated 3D structures of the wild-type and mutated proteins based on the 4DJN template. This template was the perfect candidate for the homology modeling, thanks to its high resolution of 2.2 Å and other important variables (Qmean, coverage and sequence identity). Protein PDB files were downloaded from this web server and visualized using the YASARA software (Figure 3).

### 3.6. Structural Analysis

After visualizing the structure of the wild-type and mutated proteins using YASARA, we have noticed several differences in the interactions between amino acids (hydrogen or hydrophobic bonds).

**Figure 4:**
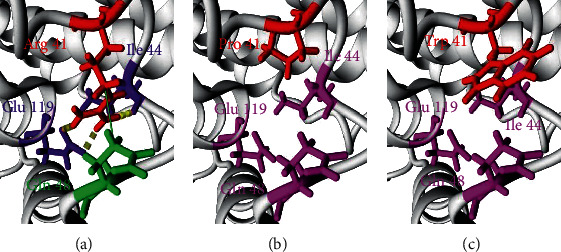
Comparison of the native RRM2B protein structure and two mutant forms. (a) The structural model of the wild-type protein (Arg 41). (b) The structural model of the first mutated form (Pro 41). (c) The structural model of the second mutated form (Trp 41). Discontinuous cylinders represent hydrogen bonds; continuous lines represent hydrophobic bonds. Red residues are the main residues where the nsSNPs appeared; purple residues are those who that have a hydrogen bond with the main residue; green residues are those who that have a hydrophobic bond with the main residue; magenta residues mark the loss of a bond between an amino acid and the main residue in the variant form that existed in the wild-type form.

As illustrated in Figure 4(a), the Arg amino acid in position 41 of the native form of *RRM2B* protein that has a hydrophobic bond with Gln 48 and hydrogen bonds with Ile 44 and Glu 119 lost interactions after being replaced by Pro (Figure 4(b)) or by Trp (Figure 4(c)).

**Figure 5:**
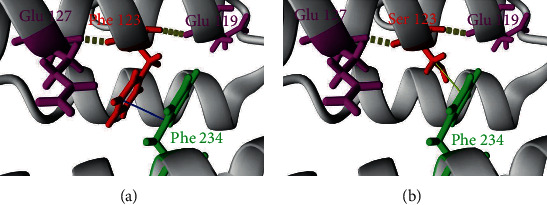
Comparison of the native RRM2B protein structure and a mutant form. (a) The structural model of the wild-type protein (Phe 123). (b) The structural model of the mutated form (Ser 123).

In the wild-type protein, the Phe 123 residue kept his 2 hydrogen bonds with Gln 127 and Glu 119 (Figure 5(a)) and his hydrophobic bond with Phe 234 when replaced by Ser (Figure 5(b)). Amino acid interaction analysis for all studied nsSNPs is summarized in Table 4, and their corresponding illustrations can be found in the supplementary materials.

Mutated proteins were superimposed with the wild type to calculate the root-mean-square deviation (RMSD). For all structures, the RMSD (Å) values did not deviate significantly from the native protein (Table 5).

## 4. Discussion

Pathogenic variants affecting *RRM2B* protein structural properties can lead to remarkable mtDNA disruption whether on the qualitative (accumulation of multiple mtDNA deletions) or quantitative (depletion of mtDNA copy number) level, causing the appearance of serious phenotypes.

In order to understand the molecular origin of these phenotypes, experimental validation seems to be crucial, although these methods require both time and resource investment. In this regard, computational tools may be an intelligent way to do this kind of studies, through the easy and open access to different software and servers dedicated to the extraction of useful information about the impact of mutations, these tools can orientate the experimental studies in the future to be more efficient and precise. Through this study, we attempted to investigate the effect of deleterious nsSNPs on the structure of the *RRM2B* protein, via different bioinformatic tools. After using a total of 13 algorithms, only nsSNPs jugged deleterious were taken into consideration allowing us to move from 8464 SNPs to 19 deleterious nsSNPs: eight SNPs in exon 6 (R186G, E194G, E194K, G195R, G200E, F206V, G216R, and D227V), 7 in exon 4 (R110P, F123S, Y124C, E131A, E131K, S139R, and I142T), 2 in exon 7 (H231Q and A235T), and 2 in exon 2 (R41W and R41P).

A stability analysis was then conducted showing that only 1 nsSNP (Y124C) increased the stability of the RRM2B protein according to I-Mutant prediction, while for the Mupro tool, all the 19 nsSNPs decreased the stability. For the conservation analysis, the results have shown that the RRM2B protein is predicted to have a total of 225 conserved residues and that 16 out of the analyzed nsSNPs are localized in very highly conserved regions; 9 SNPs represent exposed and functional residues, while 7 SNPs represent buried and structural residues. These results may lead us to suspect that most of these variants may cause important alterations of the RRM2B protein.

Our results have shown that the majority of variants are responsible for important impacts on the protein structure and amino acid interactions. Only one nsSNPs (F123S) had no impact on the amino acid interactions; these results cannot exclude the fact that this variant may have an important effect on the protein structure, since it was predicted to be deleterious, stability decreasing, and located in a highly conserved region.

A variety of experimental trials concerning the *RRM2B* gene have shown that some of the 19 nsSNPs concerned by this study are responsible for serious manifestations within subjects of various ages and phenotypes. Mutation p.Arg41Trp was found in a patient with PEO, ptosis, migraine, neck flexion weakness (mild), and proximal muscle weakness, the age of onset was 60, and the patient died at the age of 66. While the heterozygous mutation p.Arg186Gly was found along with the p.Thr218Ile mutation in a 43-year-old patient with Severe PEO, asymmetrical ptosis, proximal and distal muscle weakness, ataxia, SNHL (sensorineural hearing loss), facial weakness, low BMI (Body Mass Index), leukoencephalopathy, and depression, with an age of onset of 11 years old, another patient with Severe PEO, ptosis, SNHL, dysphonia, proximal muscle weakness, ataxia, cataracts, glaucoma, and IHD (ischemic heart disease) had the p.Gly195Arg mutation, with an age of onset of 50 years old [15].

On the other hand, Acham-Roschitz et al. reported a case of a baby girl of 8 weeks that suffered from muscular hypotonia with lack of head control as well as congenital deafness, nephrocalcinosis, and Fanconi syndrome. The genetic analysis revealed the presence of the p.Phe123Ser mutation [7]. The p.Glu194Lys mutation was reported in two siblings, a boy that showed trunk hypotonia and tubulopathy shortly after birth and died at the age of 2 months after status epilepticus and his sister that died at the same age and showed neonatal hyperlactatemia in addition to some similar complications as her brother. This mutation was also found in a baby girl that showed hypotonia soon after birth and developed respiratory distress, hyperlactatemia, and tubulopathy at an age of 3 months then died at the age of 4 months [5]. Finally, according to the study of Pitceathly et al., the p.Glu131Lys mutation was found along with p.Arg41Gln mutation in a patient with KSS syndrome [13].

Many studies are aimed at explaining the mechanism of these diseases as well as the functional consequence of their corresponding variants. Actually, the p.Arg41Trp mutation is predicted to prevent salt bridge formation. This bridge has an important role in conformation change that controls iron binding; this prediction was based on previous molecular modeling studies showing that p.Arg41Gln prevents salt bridge formation [13, 33].

As for mutations in the Arg 186 residue, it is demonstrated that they might be responsible for protein folding efficiency reduction leading to autosomal recessive disease, due to the presence of the residue at the end of the *α*-helix [15].

Likewise, Gly 195 substitution impacts some of its neighboring amino acids that contribute to iron atom coordination; this disruption is associated with adult-onset chronic progressive external ophthalmoplegia (CPEO)/multiple mtDNA deletion disorders [13].

All this experimental evidence correlates strongly with the results obtained from the bioinformatic study that we have conducted, thus proving and explaining the effect of deleterious nsSNPs on the *RRM2B* gene.

## 5. Conclusion

In the current study, we identified 19 nonsynonymous single nucleotide polymorphisms based on different computational prediction tools. To investigate the structural impact of these nsSNPs, molecular modeling analyses were done. A potential impact on protein stability was observed in 18 mutated structures in comparison with native protein. In addition, 18 variants may affect hydrogen bond and hydrophobic interactions. Our results allow for a better understanding of the effect of *RRM2B* gene nsSNPs. Therefore, these SNPs should be considered important candidates in the genetic screening of neurological diseases, such as PEO and encephalomyopathy.

## Figures and Tables

**Table 1:** Software prediction and scores for the 19 deleterious nsSNP of the *RRM2B* gene. For most prediction software: D: deleterious. Mutation Assessor: H: high. Mutation Taster: D: disease causing and A: disease causing automatic.

dbSNP ID	SNP	SIFT	POLYPHENE	LRT	M-CAP	PhD-SNP	SNAP	Mutation Assessor	Mutation taster	PROVEAN	Condel	PredictSNP	MAPP	PANTHER
rs515726181	R41W	D (0)	D (0.999)	D	D	D (0.875)	D (0.805)	H	D	D	D (0.935)	D (0.869)	D (0.426)	D (0.780)
rs200273673	R41P	D (0)	D (0.997)	D	D	D (0.885)	D (0.848)	H	D	D	D (0.911)	D (0.869)	D (0.560)	D (0.718)
rs267607025	R110P	D (0)	D (0.992)	D	D	D (0.858)	D (0.622)	H	D	D	D (0.892)	D (0.869)	D (0.588)	D (0.718)
rs515726187	F123S	D (0)	D (1)	D	D	D (0.817)	D (0.622)	H	D	D	D (0.945)	D (0.869)	D (0.409)	D (0.714)
rs1211197364	Y124C	D (0)	D (1)	D	D	D (0.858)	D (0.805)	H	D	D	D (0.945)	D (0.869)	D (0.588)	—
rs1185904532	E131A	D (0)	D (1)	D	D	D (0.875)	D (0.848)	H	D	D	D (0.945)	D (0.869)	D (0.633)	D (0.661)
rs515726188	E131K	D (0,01)	D (0.999)	D	D	D (0.885)	D (0.848)	H	D	D	D (0.896)	D (0.869)	D (0.483)	D (0.483)
rs1313813193	S139R	D (0)	D (1)	D	D	D (0.858)	D (0.848)	H	D	D	D (0.945)	D (0.869)	D (0.571)	—
rs1169870960	I142T	D (0,02)	D (0.992)	D	D	D (0.608)	D (0.555)	H	D	D	D (0.831)	D (0.869)	D (0.657)	D (0.661)
rs515726190	R186G	D (0)	D (0.997)	D	D	D (0.875)	D (0.720)	H	D	D	D (0.911)	D (0.869)	D (0.573)	—
rs515726191	E194G	D (0)	D (0.994)	D	D	D (0.817)	D (0.805)	H	D	D	D (0.897)	D (0.869)	D (0.571)	—
rs121918308	E194K	D (0)	D (0.996)	D	D	D (0.875)	D (0.848)	H	A	D	D (0.906)	D (0.869)	D (0.462)	—
rs515726192	G195R	D (0,02)	D (0.998)	D	D	D (0.875)	D (0.805)	H	D	D	D (0.857)	D (0.869)	D (0.633)	D (0.742)
rs863224192	G200E	D (0)	D (0.998)	D	D	D (0.885)	D (0.848)	H	D	D	D (0.919)	D (0.869)	D (0.877)	—
rs1283028277	F206V	D (0)	D (0.912)	D	D	D (0.817)	D (0.6221)	H	D	D	D (0.808)	D (0.869)	D (0.462)	D (0.687)
rs575109470	G216R	D (0)	D (1)	D	D	D (0.885)	D (0.720)	H	D	D	D (0.945)	D (0.869)	D (0.761)	—
rs1422185855	D227V	D (0)	D (1)	D	D	D (0.875)	D (0.805)	H	D	D	D (0.945)	D (0.869)	D (0.775)	—
rs772868983	H231Q	D (0)	D (0.998)	D	D	D (0.858)	D (0.848)	H	D	D	D (0.919)	D (0.869)	D (0.509)	D (0.687)
rs1215473912	A235T	D (0)	D (0.96)	D	D	D (0.676)	D (0.555)	H	D	D	D (0.848)	D (0.869)	D (0.633)	D (0.611)

**Table 2:** Stability software predictions and DDG scores for the 19 deleterious nsSNPs of the *RRM2B* gene.

dbSNP ID	SNP	I-Mutant		Mupro	
		Prediction	DDG value	Prediction	DDG value
rs515726181	R41W	Decrease	-0.57	Decrease	-1.809
rs200273673	R41P	Decrease	-1.06	Decrease	-2.19
rs267607025	R110P	Decrease	-1.47	Decrease	-1.312
rs515726187	F123S	Decrease	-2.49	Decrease	-1.688
rs1211197364	Y124C	Increase	0.56	Decrease	-0.935
rs1185904532	E131A	Decrease	-0.04	Decrease	-1.24
rs515726188	E131K	Decrease	-0.63	Decrease	-1.644
rs1313813193	S139R	Decrease	-0.75	Decrease	-0.849
rs1169870960	I142T	Decrease	-2.42	Decrease	-3.633
rs515726190	R186G	Decrease	-1.91	Decrease	-1.33
rs515726191	E194G	Decrease	-2.61	Decrease	-1.121
rs121918308	E194K	Decrease	-1.5	Decrease	-0.523
rs515726192	G195R	Decrease	-0.82	Decrease	-0.393
rs863224192	G200E	Decrease	-0.94	Decrease	-0.399
rs1283028277	F206V	Decrease	-2.2	Decrease	-1.157
rs575109470	G216R	Decrease	-1.08	Decrease	-0.503
rs1422185855	D227V	Decrease	-0.92	Decrease	-0.507
rs772868983	H231Q	Decrease	-0.62	Decrease	-0.199
rs1215473912	A235T	Decrease	-1.63	Decrease	-1.261

**Table 3:** Number of conserved residues in the RRM2B protein provided by the SCR prediction server.

Sequence length	351
Structural conservation cutoff	0.74
Residues predicted to be conserved	225

**Table 4:** Analysis of nsSNP effect on hydrogen bonds and hydrophobic interactions.

dbSNP ID	SNP	Hydrogen bonds		Hydrophobic bonds	
		Wild type	Variant	Wild type	Variant
rs515726181	R41P	Ile 44 and Glu 119 (2)	—	Gln 48	—
rs200273673	R41W	Ile 44 and Glu 119 (2)	—	Gln 48	—
rs267607025	R110P	Glu 114, Asp 178, Ile 176, and Asn 106	Glu 114	Glu 109	Ile 176 and Phe 111
rs515726187	F123S	Glu 119 and Gln 127	Glu 119 and Gln 127	Phe 234	Phe 234
rs1211197364	Y124C	Ile 128 and Ala 120	Ile 128 and Ala 120	Ser 112, Leu 107, Phe 111, and Phe 234	Ser 112
rs1185904532	E131A	Gln 127 (2) and Ser 135	Gln 127 and Ser 135	Gln 127	—
rs515726188	E131K	Gln 127 (2) and Ser 135	Gln 127 and Ser 135	Gln 127	Asp 100, Val 103, and His 231
rs1313813193	S139R	Ser 135, Asp 143, and Phe 45	Ser 135 and Asp 143	Phe 45	Phe 45 and Ile 44
rs1169870960	I142T	Tyr 138 and Ile 146	Tyr 138 and Ile 146	Phe 156, Ala 97, and Leu 93	Phe 156
rs515726190	R186G	Glu 114 (2), Phe 190, Thr 182, and Ser 181	Phe 190 and Thr 182	Phe 111	—
rs515726191	E194G	Phe 190	Phe 190	—	Phe 198
rs121918308	E194K	Phe 190	Phe 190 (2)	—	His 231
rs515726192	G195R	Ala 191	Ala 191 and Cys 232	—	Val 289
rs863224192	G200E	Ala 204	Ala 204	—	Ile 286
rs1283028277	F206V	Phe 202 and Lys 210	Phe 202 and Lys 210	Phe 311, Met 312, and Glu 222	Phe 311
rs575109470	G216R	Asp 74 and Ser 220 (2)	Asp74 and Ser 220 (2)	Leu 71	—
rs1422185855	D227V	Leu 223, Arg 226 (2), Trp 64, and His 231	Leu 223 and His 231	—	His 231
rs772868983	H231Q	Asp 227 and Ala 235	Asp 227 and Ala 235	Gln 127	—
rs1215473912	A235T	His 231 and Phe 239	His 231 and Phe 239	Ala 191 and Val 187	Ala 191

(2): two interactions with the same residue.

**Table 5:** Root-mean-square deviation (RMSD) of deleterious nsSNPs.

dbSNP ID	SNP	RMSD (Å)
rs515726181	R41W	0.0274
rs200273673	R41P	0.0236
rs267607025	R110P	0.0446
rs515726187	F123S	0.0032
rs1211197364	Y124C	0.0019
rs1185904532	E131A	0.0165
rs515726188	E131K	0.0275
rs1313813193	S139R	0.0964
rs1169870960	I142T	0.0025
rs515726190	R186G	0.0114
rs515726191	E194G	0.0160
rs121918308	E194K	0.0239
rs515726192	G195R	0.0378
rs863224192	G200E	0.0537
rs1283028277	F206V	0.0059
rs575109470	G216R	0.0684
rs1422185855	D227V	0.0101
rs772868983	H231Q	0.0181
rs1215473912	A235T	0.0251

## Data Availability

All data used in this study are included within the article and supplementary information file.

## Supplementary Materials

Supplementary Materials Figure S1: comparison of the native RRM2B protein structure and a mutant form. (A) The structural model of the wild-type protein (Arg 110). (B) The structural model of the mutated form (Pro 110). Figure S2: comparison of the native RRM2B protein structure and a mutant form. (A) The structural model of the wild-type protein (Tyr 124). (B) The structural model of the mutated form (Cys 124). Figure S3: comparison of the native RRM2B protein structure and two mutant forms. (A) The structural model of the wild-type protein (Glu 131). (B) The structural model of the first mutated form (Ala 131). (C) The structural model of the second mutated form (Lys 131). Figure S4: comparison of the native RRM2B protein structure and a mutant form. (A) The structural model of the wild-type protein (Ser 139). (B) The structural model of the mutated form (Arg 139). Figure S5: comparison of the native RRM2B protein structure and a mutant form. (A) The structural model of the wild-type protein (Ile 142). (B) The structural model of the mutated form (Thr 142). Figure S6: comparison of the native RRM2B protein structure and a mutant form. (A) The structural model of the wild-type protein (Arg 186). (B) The structural model of the mutated form (Gly 186). Figure S7: comparison of the native RRM2B protein structure and two mutant forms. (A) The structural model of the wild-type protein (Glu 194). (B) The structural model of the first mutated form (Gly 194). (C) The structural model of the second mutated form (Lys 194). Figure S8: comparison of the native RRM2B protein structure and a mutant form. (A) The structural model of the wild-type protein (Gly 195). (B) The structural model of the mutated form (Arg 195). Figure S9: comparison of the native RRM2B protein structure and a mutant form. (A) The structural model of the wild-type protein (Gly 200). (B) The structural model of the mutated form (Glu 200). Figure S10: comparison of the native RRM2B protein structure and a mutant form. (A) The structural model of the wild-type protein (Phe 206). (B) The structural model of the mutated form (Val206). Figure S11: comparison of the native RRM2B protein structure and a mutant form. (A) The structural model of the wild-type protein (Gly 216). (B) The structural model of the mutated form (Arg 216). Figure S12: comparison of the native RRM2B protein structure and a mutant form. (A) The structural model of the wild-type protein (Asp 227). (B) The structural model of the mutated form (Val 227). Figure S13: comparison of the native RRM2B protein structure and a mutant form. (A) The structural model of the wild-type protein (His 231). (B) The structural model of the mutated form (Gln 231). Figure S14: comparison of the native RRM2B protein structure and a mutant form. (A) The structural model of the wild-type protein (Ala 235). (B) The structural model of the mutated form (Thr 235).
