# Supplementary material for: Prediction of the Impact of Deleterious Nonsynonymous Single Nucleotide Polymorphisms on the Human RRM2B Gene: A Molecular Modeling Study
Source: Biomed Res Int. 2020 Jul 25;2020:7614634. doi: 10.1155/2020/7614634 (PMC7399733; doi:10.1155/2020/7614634)
Supplement: Supplementary Materials — Figure S1: comparison of the native RRM2B protein structure and a mutant form. (A) The structural model of the wild-type protein (Arg 110). (B) The structural model of the mutated form (Pro 110). Figure S2: comparison of the native RRM2B protein structure and a mutant form. (A) The structural model of the wild-type protein (Tyr 124). (B) The structural model of the mutated form (Cys 124). Figure S3: comparison of the native RRM2B protein structure and two mutant forms. (A) The structural model of the wild-type protein (Glu 131). (B) The structural model of the first mutated form (Ala 131). (C) The structural model of the second mutated form (Lys 131). Figure S4: comparison of the native RRM2B protein structure and a mutant form. (A) The structural model of the wild-type protein (Ser 139). (B) The structural model of the mutated form (Arg 139). Figure S5: comparison of the native RRM2B protein structure and a mutant form. (A) The structural model of the wild-type protein (Ile 142). (B) The structural model of the mutated form (Thr 142). Figure S6: comparison of the native RRM2B protein structure and a mutant form. (A) The structural model of the wild-type protein (Arg 186). (B) The structural model of the mutated form (Gly 186). Figure S7: comparison of the native RRM2B protein structure and two mutant forms. (A) The structural model of the wild-type protein (Glu 194). (B) The structural model of the first mutated form (Gly 194). (C) The structural model of the second mutated form (Lys 194). Figure S8: comparison of the native RRM2B protein structure and a mutant form. (A) The structural model of the wild-type protein (Gly 195). (B) The structural model of the mutated form (Arg 195). Figure S9: comparison of the native RRM2B protein structure and a mutant form. (A) The structural model of the wild-type protein (Gly 200). (B) The structural model of the mutated form (Glu 200). Figure S10: comparison of the native RRM2B protein structure and a mutant form. (A) The stru [file 7614634.f1.pdf]

Supplementary materials:

Description: Yellow discontinuous cylinders: represent hydrogen bonds, continuous lines: represent hydrophobic bonds. Red residues: are the main residues where the nsSNPs appeared; Purple residues: are those that have a hydrogen bond with the main residue; Green residues: are those that have a hydrophobic bond with the main residue; Orange residues: are those that have both hydrogen and hydrophobic bonds with the main residue; Blue residues: mark the addition of a new bond between a new amino acid and the main residue in the variant form that never existed in the wild type form; Magenta residues: mark the loss of a bond between an amino acid and the main residue in the variant form that existed in the wild type form.

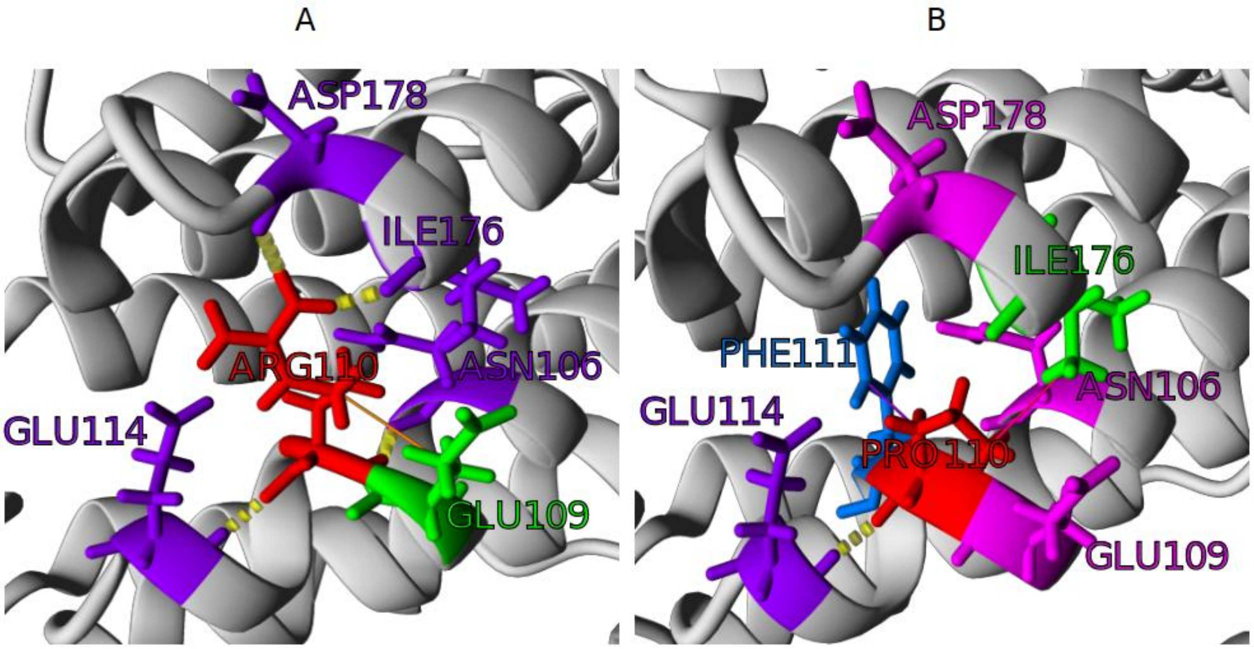

Figure S1: Comparison of the native RRM2B protein structure and a mutant form. (A) the structural model of the wild type protein (Arg110). (B) the structural model of the mutated form (Pro110).

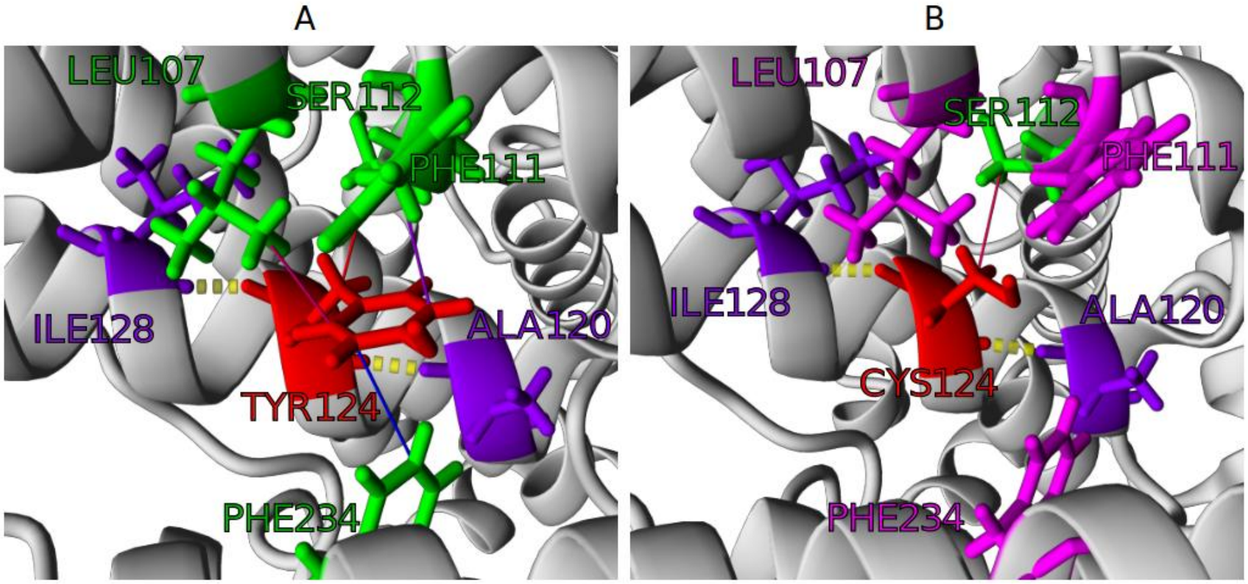

Figure S2: Comparison of the native RRM2B protein structure and a mutant form. (A) the structural model of the wild type protein (Tyr124). (B) the structural model of the mutated form (Cys124).

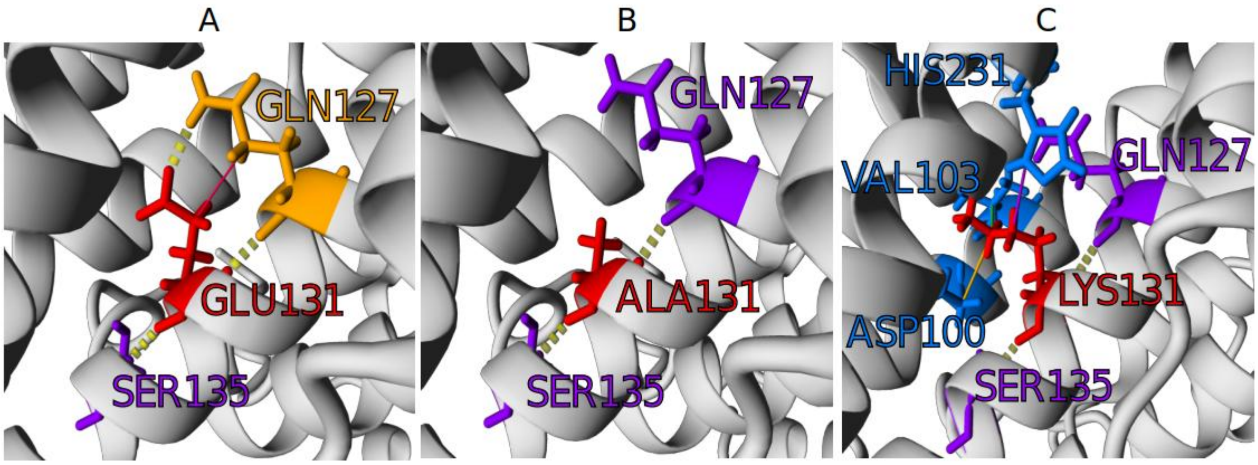

Figure S3: Comparison of the native RRM2B protein structure and two mutant forms. (A) the structural model of the wild type protein (Glu131). (B) the structural model of the first mutated form (Ala131). (C) the structural model of the second mutated form (Lys131).

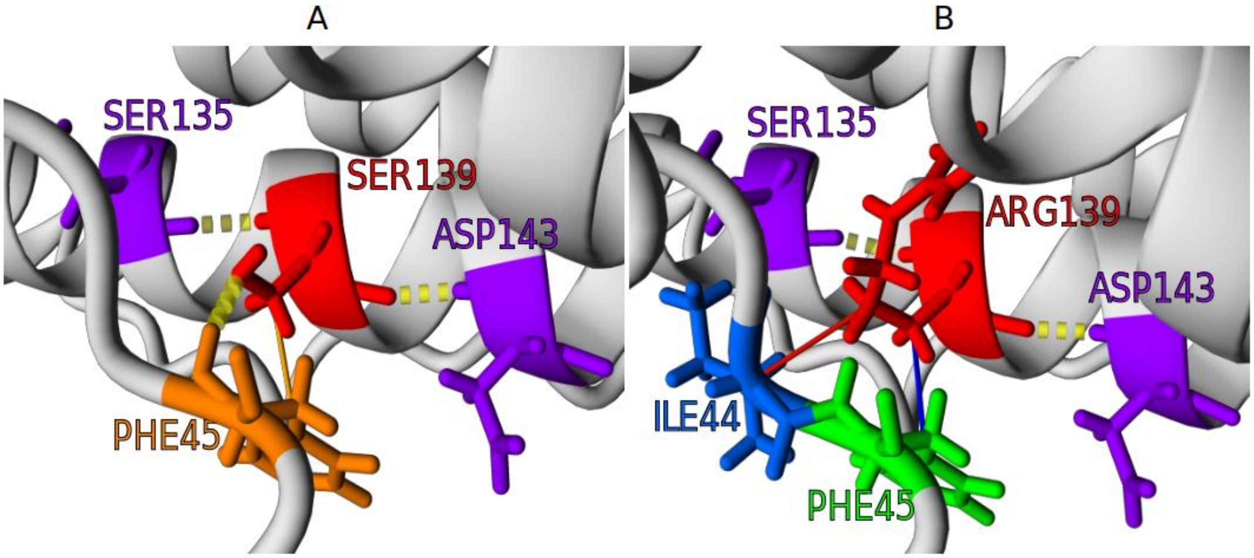

Figure S4: Comparison of the native RRM2B protein structure and a mutant form. (A) the structural model of the wild type protein (Ser139). (B) the structural model of the mutated form (Arg139).

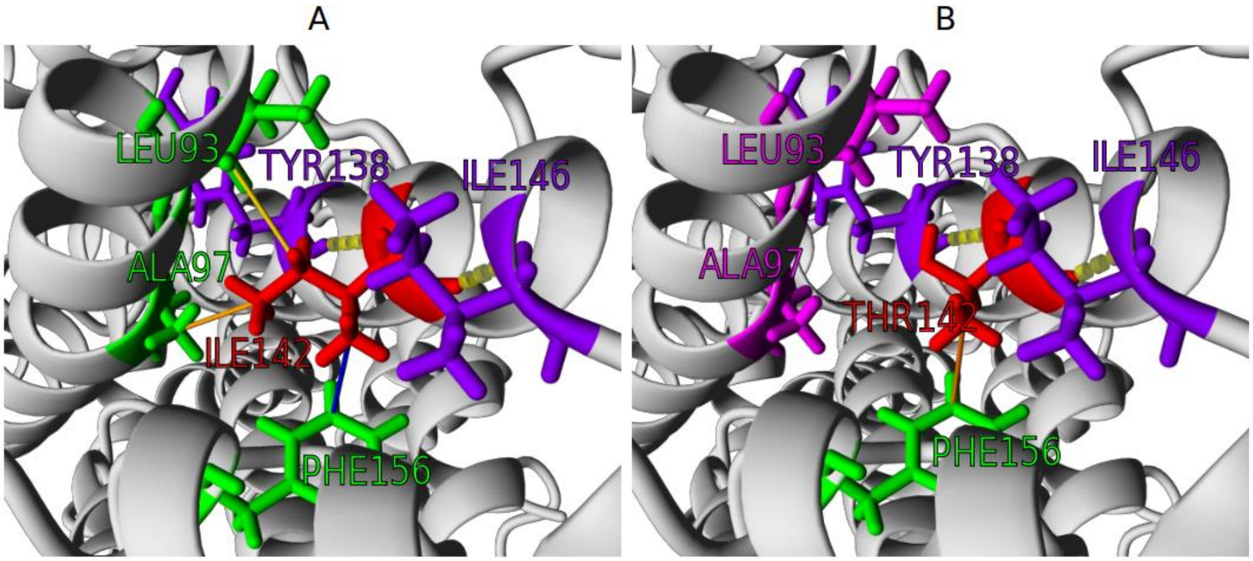

Figure S5: Comparison of the native RRM2B protein structure and a mutant form. (A) the structural model of the wild type protein (Ile142). (B) the structural model of the mutated form (Thr142).

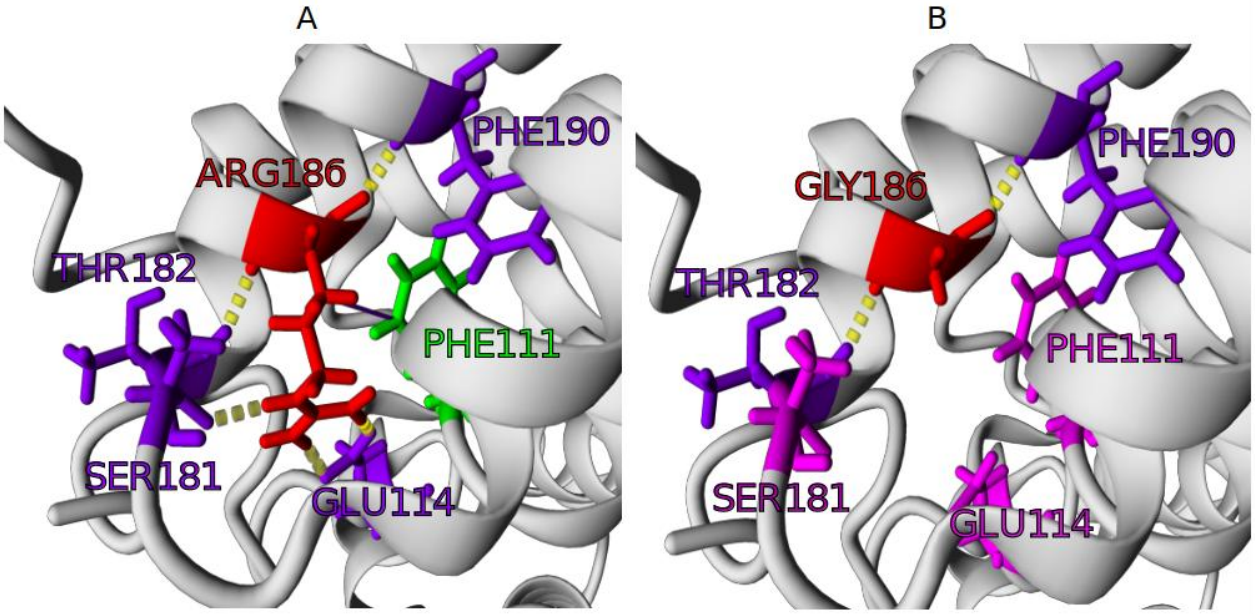

Figure S6: Comparison of the native RRM2B protein structure and a mutant form. (A) the structural model of the wild type protein (Arg186). (B) the structural model of the mutated form (Gly186).

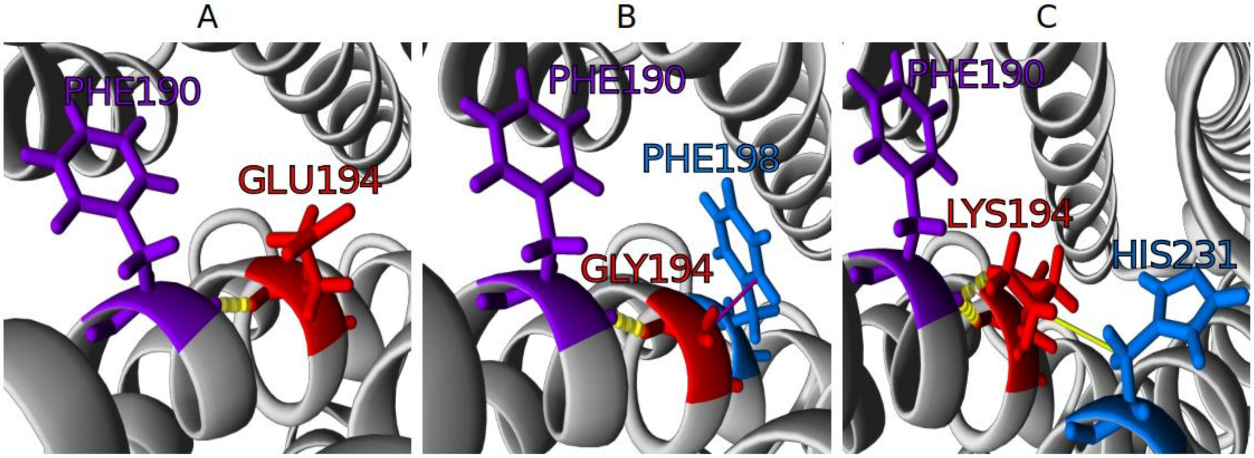

Figure S7: Comparison of the native RRM2B protein structure and two mutant forms. (A) the structural model of the wild type protein (Glu194). (B) the structural model of the first mutated form (Gly194). (C) the structural model of the second mutated form (Lys194).

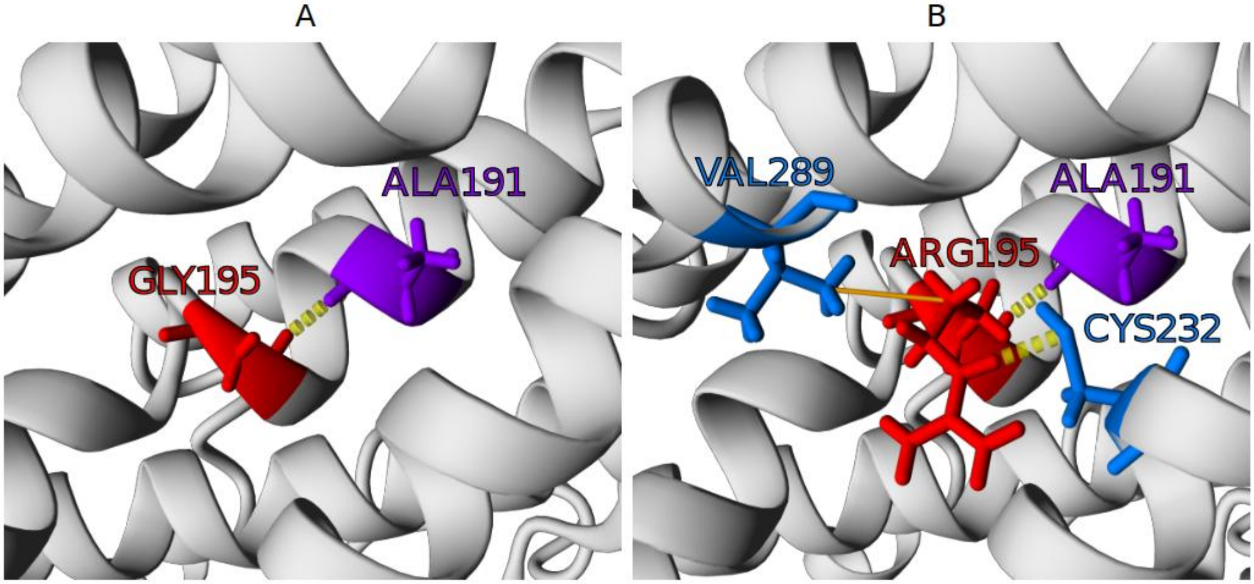

Figure S8: Comparison of the native RRM2B protein structure and a mutant form. (A) the structural model of the wild type protein (Gly195). (B) the structural model of the mutated form (Arg195).

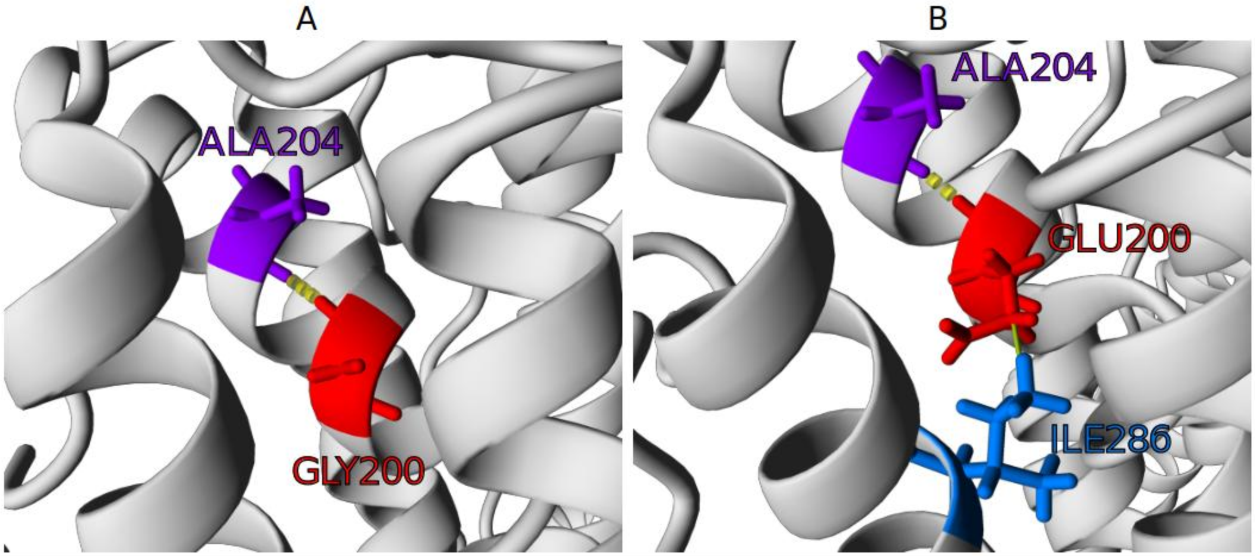

Figure S9: Comparison of the native RRM2B protein structure and a mutant form. (A) the structural model of the wild type protein (Gly200). (B) the structural model of the mutated form (Glu200).

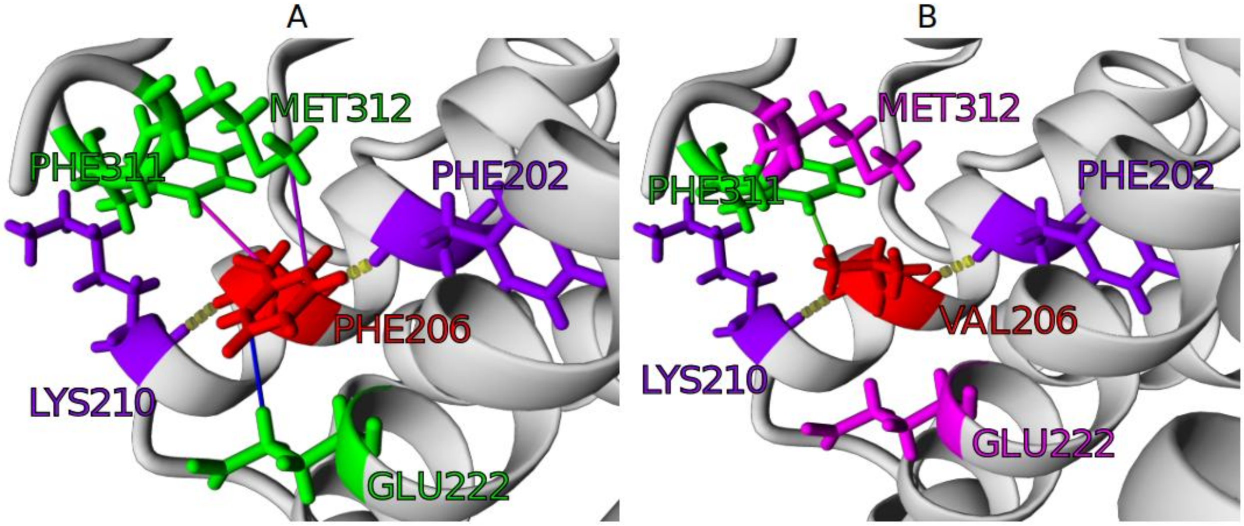

Figure S10: Comparison of the native RRM2B protein structure and a mutant form. (A) the structural model of the wild type protein (Phe206). (B) the structural model of the mutated form (Val206).

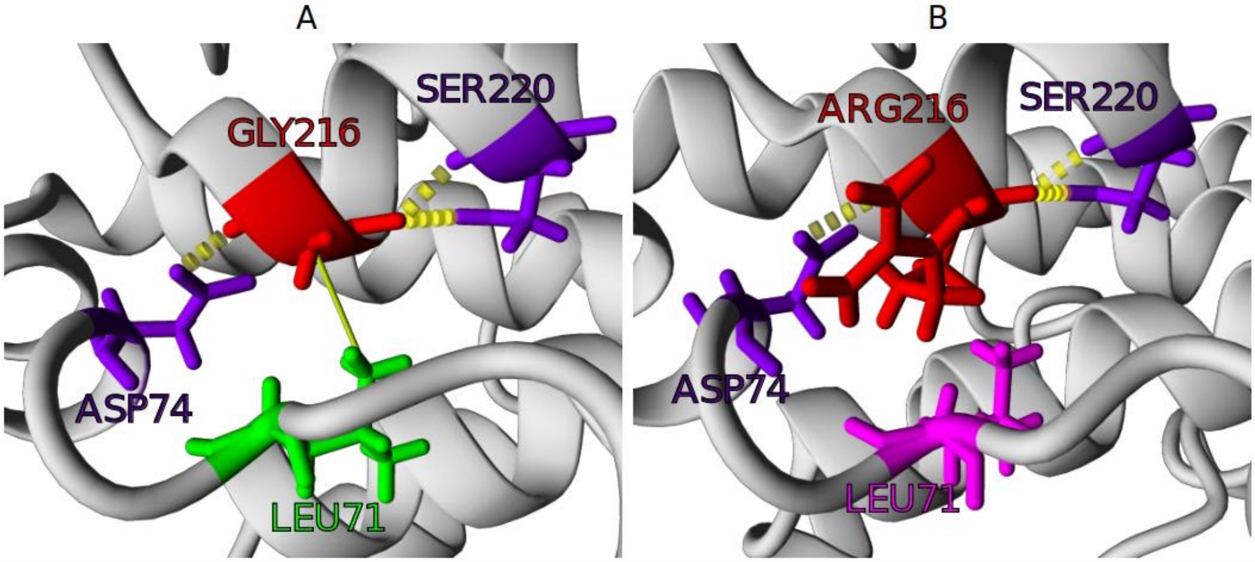

Figure S11: Comparison of the native RRM2B protein structure and a mutant form. (A) the structural model of the wild type protein (Gly216). (B) the structural model of the mutated form (Arg216).

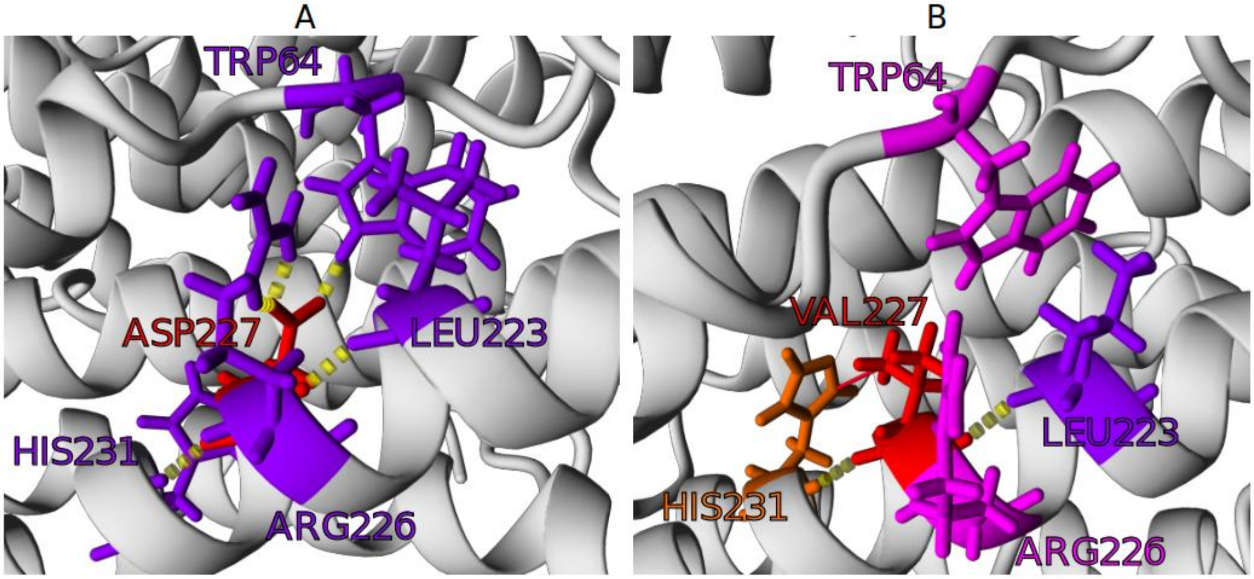

Figure S12: Comparison of the native RRM2B protein structure and a mutant form. (A) the structural model of the wild type protein (Asp227). (B) the structural model of the mutated form (Val227).

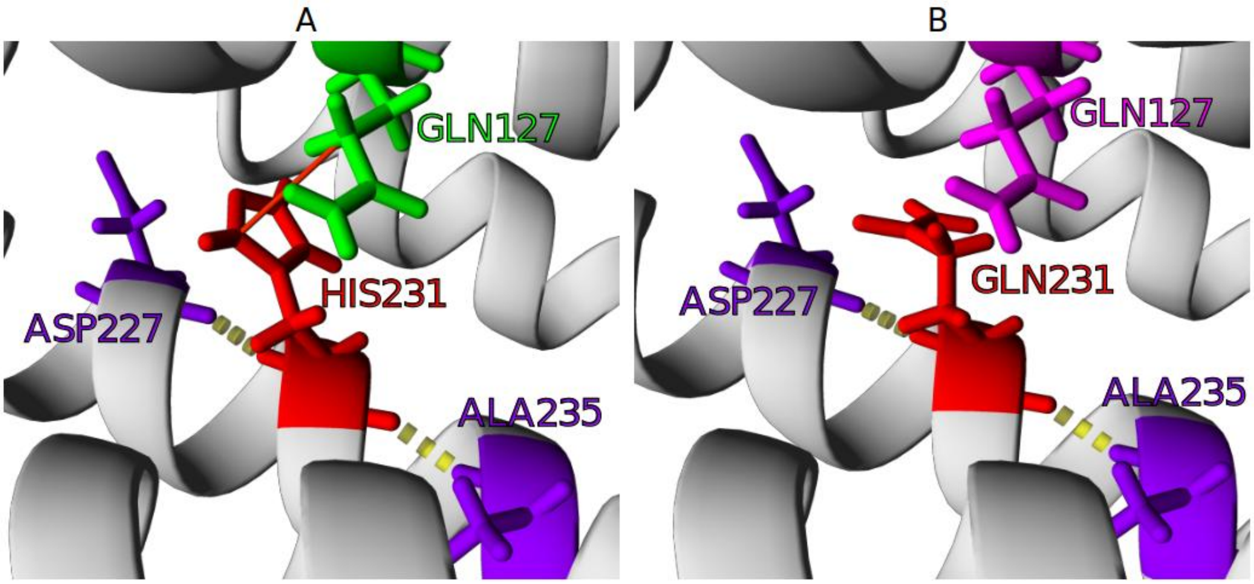

Figure S13: Comparison of the native RRM2B protein structure and a mutant form. (A) the structural model of the wild type protein (His231). (B) the structural model of the mutated form (Gln231).

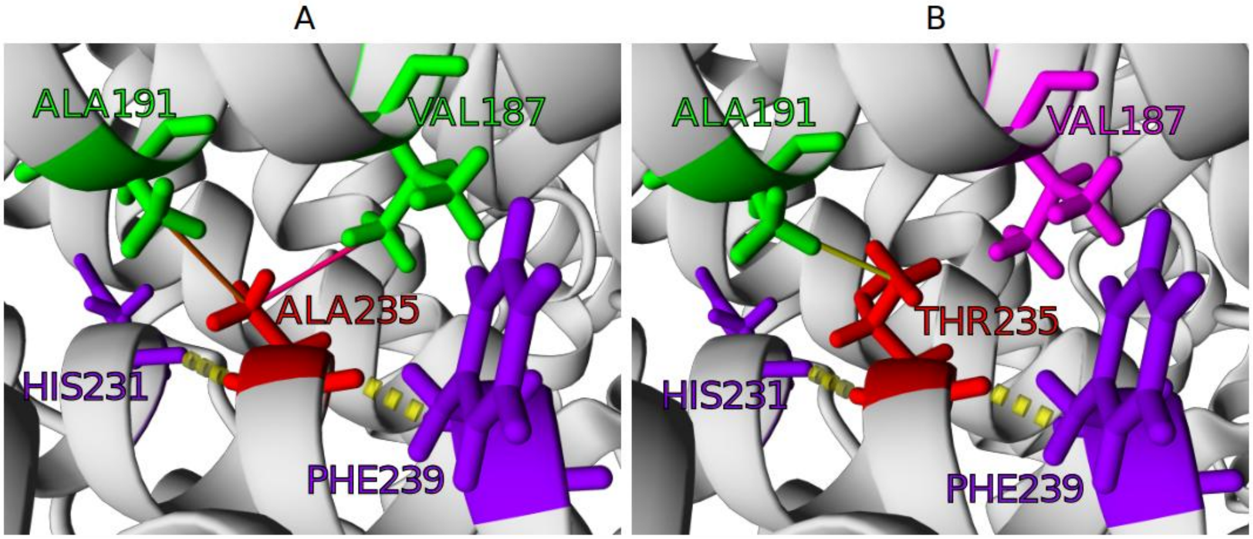

Figure S14: Comparison of the native RRM2B protein structure and a mutant form. (A) the structural model of the wild type protein (Ala235). (B) the structural model of the mutated form (Thr235).
